# Prevalence of depression, anxiety and burnout in medical students at the University of Namibia

**DOI:** 10.4102/sajpsychiatry.v29i0.2044

**Published:** 2023-05-31

**Authors:** Nelao T. Mhata, Vuyokazi Ntlantsana, Andrew M. Tomita, Kissah Mwambene, Shamima Saloojee

**Affiliations:** 1 Discipline of Psychiatry, School of Clinical Medicine, College of Health Sciences, University of KwaZulu-Natal, Durban, South Africa; 2 Discipline of Psychiatry, KwaZulu-Natal Research Innovation and Sequencing Platform (KRISP), College of Health Sciences, University of KwaZulu-Natal, Durban, South Africa; 3 Discipline of Psychiatry, Centre for Rural Health, School of Nursing and Public Health, College of Health Sciences, University of KwaZulu-Natal, Durban, South Africa; 4 Department of Psychiatry, School of Medicine, Faculty of Health Sciences and Veterinary Medicine, University of Namibia, Windhoek, Namibia; 5 Mental Health Centre, Windhoek Central Hospital, Windhoek, Namibia

**Keywords:** anxiety, depression, burnout, medical students, Namibia

## Abstract

**Background:**

There is an increased prevalence of depression, anxiety, and burnout among medical students worldwide with no information from Namibia.

**Aim:**

This study aimed to determine the prevalence and factors associated with depression, anxiety, and burnout among medical students at the University of Namibia (UNAM).

**Methods:**

A quantitative descriptive cross-sectional survey was conducted utilising a specially designed questionnaire for the study and standardised instruments to evaluate depression, anxiety, and burnout.

**Results:**

Of the 229 students in this study, 71.6% were female and 28.4% were male. The prevalence of depression, anxiety, and burnout was 43.6%, 30.6%, and 36.2%, respectively. The prevalence of emotional exhaustion (EX), cynicism (CY), and professional efficacy (EF) was 68.1% (*n* = 156), 77.3% (*n* = 177) and 53.3% (*n* = 122), respectively. In the final regression model, participants with a current psychiatric illness were more likely to screen positive for depression (adjusted odds ratio [aOR] 4.06, confidence interval [CI] 1.28–12.91; *p* = 0.02) and anxiety (aOR: 3.63, CI: 1.17–11.23; *p* = 0.03). Emotional exhaustion and cynicism were significantly associated with female gender (EX: aOR, 0.40, CI: 0.20–0.79; *p* = 0.01) (CY: aOR, 0.42, CI: 0.20–0.91; *p* = 0.03).

**Conclusion:**

More than one in three medical students at the UNAM were either depressed or burnt out.

**Contribution:**

This is the first study to highlight the mental health needs of medical students at the University of Namibia.

## Introduction

About one in three medical students globally experiences depression, anxiety, or burnout.^1,2^ There is evidence in the literature that medical students have a higher prevalence of depression,^3,4,5,6^ anxiety,^1,3,4,7^ burnout^2,8,9^ and overall psychological distress^4^ than do individuals in the general population.^4,5^ This is likely because medical students are exposed to multiple personal and organisational stressors during their training.^4^ Previous studies have found that medical students are exposed to academically demanding learning environments^3,4^ and patient suffering.^10^ Furthermore, several studies conducted during and after the recent global coronavirus disease 2019 (COVID-19) pandemic showed that medical students were a high-risk group for mental health problems.^11^ The distress medical students experience manifests as depression, anxiety, and burnout.^3,4^

A systematic review conducted in 2019 estimated that the global pooled prevalence of depression in medical students was 27%, with studies from Africa having the highest pooled prevalence rates at 40.9%.^6^ Some of the factors associated with depression in medical students in the literature include female gender,^12^ having a history of psychological or psychiatric treatment,^13^ the first 2 years of medical school^7^ and experiencing a stressful life event.^7^ Although medical students have higher rates of depression, they are not more likely than the general population to seek treatment for depression. Reasons for not seeking treatment timeously include a lack of time, fears regarding confidentiality, the stigma associated with a mental illness, the cost of seeking care, and implications for academic standing.^14^

Anxiety, although almost as common as depression has received less attention in the literature.^15^ As with depressive disorders, anxiety disorders are often not detected and treated.^1,15^ Anxiety and depression also tend to occur co-morbidly.^7,12^ A systematic review conducted in 2019 found that the global prevalence of anxiety among medical students was 33.8%.^1^ Factors associated with anxiety in medical students are similar to those in depression and include female gender,^7,12^ having a history of psychological or psychiatric treatment,^13^ the first 2 years of medical school,^7^ exposure to patients’ suffering, death,^10^ a perceived lack of psychological^12^ and poor social support.^7^ There is evidence of a bi-directional relationship between anxiety and academic performance. Previous studies found that high levels of anxiety were associated with poor performance in examinations and poor performance led to anxiety.^16^

Burnout is characterised by a triad of emotional exhaustion (EX), cynicism (CY) and reduced professional accomplishment related to work or caregiving activities.^17^ Previously thought to only occur in working professionals, it is evident that burnout also occurs in medical students.^2^ The exhaustion dimension correlates with workload demands and is the most widely reported dimension of burnout.^17^ Exhaustion can lead to CY and indifference. Cynicism was reported to be associated with a negative and callous attitude towards others and work.^17^ Previously found to develop through the course of medical training, CY could lead to a decline in empathy and humanitarianism.^18^ Compassion and caring for others are important traits for future medical doctors. Reduced personal accomplishment is a marker of inefficiency.^17^

A systematic review and meta-analysis reported that the global prevalence of burnout in medical students was 44.2%. However, this meta-analysis did not include information from Africa.^2^ Individual studies from Egypt (88%)^19^ and Uganda (54.5%)^8^ found a high prevalence of burnout in medical students from Africa. A study from Morocco confirmed the high prevalence of burnout among medical students from Africa, with 93% and 68% of medical students reporting exhaustion and disengagement, respectively.^20^

Burnout, depression, and anxiety can occur simultaneously, with overlapping symptomatology and aetiology.^12,21^ There is evidence that these conditions could lead to several adverse outcomes including substance abuse,^3^ academic dishonesty,^3^ dropping out of medical school,^22^ poor self-care^23^ and even suicide.^3^

Physician depression, anxiety and burnout are common worldwide,^24^ with a suggestion that the onset of physician burnout occurs during medical school.^25^ A systematic review concurred that doctors in sub-Saharan Africa have a high prevalence of burnout.^24^ It is thus important to identify and treat mental health problems that are encountered during the training of this vulnerable group. However, there is generally limited information from Africa. For example, no studies from Africa met the inclusion criteria in a meta-analysis of 183 studies from 43 countries that investigated the prevalence of depression in medical students.^5^

The University of Namibia medical school was established in 2009 and is the only medical school in the country. To the best of the authors’ knowledge there is no information regarding the prevalence of depression, anxiety, or burnout in students at the school. The aim of this study therefore was to determine the prevalence and factors associated with depression, anxiety, and burnout among medical students at the University of Namibia.

The study hypothesis was that there would be a high prevalence of depression, anxiety, and burnout among medical students from the University of Namibia. Specifically, the authors predicted that the prevalence of each of these disorders would be greater than 30%. It was expected that depression, anxiety, and burnout would be associated with female gender and the first 2 years of medical school.

## Methods

### Study design

A cross-sectional, quantitative descriptive survey was conducted at the University of Namibia.

### Study setting

The study was conducted at the School of Medicine, University of Namibia (UNAM). The UNAM is a public university located in Windhoek, the capital city of Namibia. A total of 553 students were enrolled in the Bachelor of Medicine and Bachelor of Surgery degree (MBChB) for the academic year 2022. Students at the University of Namibia are divided into pre-clinical (1–3 years) and clinical (4–6 years) groups. Pre-clinical students are based at the School of Medicine in Windhoek, while clinical students are based at teaching hospitals in Windhoek (fourth and sixth years) and Oshakati and Onandjokwe (fifth years). The Oshakati and Onandjokwe Hospital Complex is about 750 km from Windhoek. The medium of teaching at UNAM is English.

### Study population and sampling strategy

Paper-based self-administered questionnaires were handed out to 229 medical students who were present in class or at the teaching hospitals and were willing to participate in the study during May 2022. The response rate was 98%. Male and female students who were over the age of 18 were included in the study. Participation was entirely voluntary, and informed written consent was obtained from each participant. Confidentiality was maintained throughout, and students were not asked to fill in any identifying data. An information leaflet regarding depression, anxiety, and burnout was also handed out to all participants. Information on where and how to seek help for mental health problems was included.

### Measurement tools

Participants were requested to complete four self-report questionnaires: (1) a sociodemographic questionnaire that was developed by the authors specifically for the study to assess sociodemographic factors and academic and clinical-related factors; (2) the Patient Health Questionnaire-9 (PHQ-9) to assess the prevalence of depression; (3) the Generalised anxiety disorder questionnaire-7 (GAD-7) to assess the prevalence of anxiety, and (4) the Maslach Burnout Inventory general survey for students (MBI-GS [S]) also known as the Maslach Burnout Inventory Student Survey (MBI-SS) to asses for burnout.

The variables obtained on the sociodemographic questionnaire included age, gender, marital status, number of children, current year of study, monthly income of the student’s family, source of study funding, above average academic performance in the previous year (achievement of >75% is a distinction at UNAM), accommodation, parents, or siblings with a tertiary degree. Clinical data collected included current treatment for a medical illness, psychiatric illness, and substance use. In this study, current treatment refers to the treatment of a medical and psychiatric illness in the last three months (treatment and illness not specified), and current substance use refers to the use of alcohol and cannabis within the last three months. A question on COVID-19 status, past or present was also included.

The PHQ-9, a 9-item questionnaire is a screening tool for depressive symptoms. It has also been used as a diagnostic tool and a measure of the severity of depressive symptoms. Each of the nine questions is responded to on a 4-point scale, with scores ranging from 0 to 27. Scores of 5, 10, 15 and 20 represent mild, moderate, moderately severe, and severe depression, respectively. Patient Health Questionnaire-9 scores of ≥10 have been found to have a sensitivity and specificity of 88% for the diagnosis of depression.^26^ Although the PHQ-9 has not yet been validated in Namibia, it has been used in studies conducted in Namibia and was found to be a reliable tool to measure depression.^27^

The GAD-7 is a self-reported questionnaire that is used to screen and measure the severity of GAD. The GAD-7 has a set of seven questions that are scored on a 4-point scale from 0 to 3, with scores ranging from 0 to 21. Scores of 5, 10, and 15 represent mild, moderate, and severe anxiety, respectively. A cut-off score of ≥10 has a sensitivity of 0.89 and a specificity of 0.82 for identifying (GAD). The GAD-7 can also be used as a general screening tool to identify other anxiety disorders including panic disorder, post-traumatic stress disorder, and social anxiety.^15^ In this study, a cut-off score of 10 was used to identify students who screened positive for anxiety. The GAD-7 has been validated for use in low and middle-income countries (LMICs), including South Africa.^28^

The MBI is considered to be the gold standard for identifying burnout in the literature.^29^ The MBI-SS is a 15-item questionnaire that is modified from the MBI General Survey. It is designed specifically to measure student burnout,^30^ and was found to be a reliable and valid tool to identify burnout in medical students.^31^ The MBI-SS questionnaire comprises three separate scales. The EX scale (EX; 5 items) is a measure of being emotionally overextended and exhausted by one’s studies, and scores of (0–9), (10–14), and (>14) represent low, moderate, and high EX, respectively. The CY scale (CY; 4 items) is a measure of negative and cynical feelings towards one’s studies and scores of (0–1), (2–6), and (>6) represent low, moderate, and high CY, respectively. The professional efficacy (EF) scale (EF; 6 items) assesses feelings of incompetence as a student, and scores of (>27), (23–27), and (<23) represent low, moderate, and high feelings of incompetence, respectively. Low scores on the EF scale (scores < 23) and high scores on the EX scale (scores > 14) and CY scale (scores > 6) are suggestive of burnout.^32^ In this study, the overall prevalence of burnout was calculated using a three-dimensional model. Only students who scored: (1) high on the EX scale (scores > 14); (2) high on the CY scale (scores > 6); and (3) low on the EF scale (scores < 23) were considered to be suffering from burnout.

### Statistical analysis

Descriptive statistics were used to summarise the sociodemographic characteristics, the clinical characteristics, and the depression, anxiety and burnout scores from the PHQ-9, GAD-7, and MBI-SS scores, respectively. Descriptive statistics such as means, standard deviations, medians and ranges were utilised for continuous data, and frequencies and percentages were utilised for categorial data. The sociodemographic and clinical characteristics associated with depression, anxiety and burnout were analysed using bivariate analysis. The following statistical tests were used to test for the association between variables: (1) the independent samples *t*-tests to test for the association between dichotomous categorical and continuous variables; (2) one way-ANOVA (analysis of variance) for the association between categorical variables with three or more categories; and (3) continuous variables and Pearson’s chi-square test for the association between categorical variables. Multivariate linear regression was used to assess mental health outcomes controlling for sociodemographic and clinical variables. A *p* of < 0.05 was considered to be statistically significant.

### Ethical considerations

An application for full ethical approval was made to the Biomedical Research Ethics Committee (BREC) of the University of KwaZulu-Natal, the Ministry of Health and Social Services in Namibia and the University of Namibia. Full ethical consent was received on 21 March 2022. The ethics approval number is (Ref. no. BREC/00003547/2021).

## Review findings

The median age of the 229 medical students who participated in this study was 22 (interquartile range [IQR] 20.0, 23.0) years. There were 164 (71.6%) female and 65 male (28.4%) participants. Students across all 6 years of the medical school participated in this study, with the highest number of participants in their fifth year, 24% (*n* = 55). About one third of the participants, 36.3% (*n* = 81), came from families with a household income of less than N$5000.00. The majority of the students were single 97.8% (*n* = 22). The proportion of participants with a psychiatric illness and a medical illness in the last 3 months was 9.6% (*n* = 22) and 11.8% (*n* = 27), respectively. All the participants were treated for either depression or anxiety (100%), and 40% of these participants (*n* = 10) were treated for comorbid depression and anxiety. More than one-quarter of the participants, 28.7% (*n* = 64), tested positive for COVID-19 previously, while 44.1% (*n* = 101) admitted to current alcohol use and 7.0% (*n* = 16) to current cannabis use (Table 1).

**TABLE 1:** Sociodemographic information, clinical and behaviour characteristics.

Variables	*n*	%
***N***	229	-
Age, median (IQR)	22.0 (20.0, 23.0)	-
**Gender**		
Female	164	71.6
Male	65	28.4
**Marital status**		
Single	224	97.8
Married	5	2.2
**Year of study (number of students in class)**		
1 (80)	30	13.1
2 (75)	38	16.6
3 (79)	34	14.8
4 (106)	41	17.9
5 (95)	55	24.0
6 (118)	31	13.5
**Monthly income of student’s family:**		
N$0 – N$1000	37	16.6
N$1001 – N$2500	15	6.7
N$2501 – N$5000	29	13.0
N$5001 – N$9999	23	10.3
>N$10 000	119	53.4
**Academic achievement in previous year**		
Less than 75%	185	80.8
More than 75%	44	19.2
**Children**		
Has child or children	9	3.9
**Current psychiatric treatment**		
Yes	22	9.6
**Current treatment for medical illness**		
Yes	27	11.8
**Past COVID-19 diagnosis**		
Yes	64	28.7
**Current alcohol use**		
Yes	101	44.1
**Current cannabis use**		
Yes	16	7.0

N$, Namibian dollars; COVID, coronavirus disease 2019.

Note: Content in brackets is the reference or base group.

The overall prevalence of depression was 43.6% (*n* = 100), with 26.6% (*n* = 61) and 17.0% (*n* = 39) of the participants screening positive for moderate and severe depression, respectively. The overall prevalence of anxiety was 30.6% (*n* = 70), with 20.1% (*n* = 46) and 10.5% (*n* = 24) of participants screening positive for moderate and severe anxiety, respectively. By year of study, 37% (*n* = 11) of students screened positive for depression in their first year of study, 55% (*n* = 21) in the second year, and 53% (*n* = 18) in the third year of study. Similarly, the number of participants who screened positive for anxiety was 26% (*n* = 8) in the first year, 42% (*n* = 16) and 44% (*n* = 15) in the second and third years, respectively. The prevalence of anxiety was the lowest in year 6, 19% (*n* = 6). The overall prevalence of burnout was 36.2% (*n* = 83). Participants reported high levels of burnout in all dimensions. The prevalence of EX, CY and EF was 68.1% (*n* = 156), 77.3% (*n* = 177) and 53.3% (*n* = 122), respectively (Table 2).

**TABLE 2:** The overall prevalence of depression, anxiety and burnout and prevalence by year of study.

Variable	Prevalence		Year 1		Year 2		Year 3		Year 4		Year 5		Year 6		*p*
	*n*	%	*n*	%	*n*	%	*n*	%	*n*	%	*n*	%	*n*	%	
*N*	-	-	30	-	38	-	34	-	41	-	55	-	31	-	-
**Depression**	100	43.6	-	-	-	-	-	-	-	-	-	-	-	-	-
Mild	-	-	19	63	17	45	16	47	26	63	29	53	22	71	0.18
Moderate	-	-	6	20	16	42	8	24	9	22	15	27	7	23	-
Severe	-	-	5	17	5	13	10	29	6	15	11	20	2	6	-
**Anxiety**	70	30.6	-	-	-	-	-	-	-	-	-	-	-	-	-
Mild	-	-	22	73	22	58	19	56	33	80	38	69	25	81	0.16
Moderate	-	-	4	13	11	29	12	35	5	12	9	16	5	16	-
Severe	-	-	4	13	5	13	3	9	3	7	8	15	1	3	-
**Burnout**	83	36.2	-	-	-	-	-	-	-	-	-	-	-	-	-
Negative	-	-	20	67	20	53	22	65	24	59	35	64	25	81	0.26
Positive	-	-	10	33	18	47	12	35	17	41	20	36	6	19	-
**EX**	156	68.1	-	-	-	-	-	-	-	-	-	-	-	-	-
Low	-	-	7	23	6	16	6	18	10	24	14	25	6	19	0.99
Moderate	-	-	2	7	4	11	4	12	5	12	6	11	3	10	-
High	-	-	21	70	28	74	24	71	26	63	35	64	22	71	-
**Cynicism**	177	77.3	-	-	-	-	-	-	-	-	-	-	-	-	-
Low	-	-	0	0	0	0	0	0	1	2	3	5	0	0	0.44
Moderate	-	-	8	27	5	13	9	26	10	24	10	18	6	19	-
High	-	-	22	73	33	87	25	74	30	73	42	76	25	81	-
**EF**	122	53.3	-	-	-	-	-	-	-	-	-	-	-	-	-
Low	-	-	13	43	22	58	16	47	28	68	32	58	11	35	0.02
Moderate	-	-	5	17	10	26	7	21	3	7	8	15	13	42	-
High	-	-	12	40	6	16	11	32	10	24	15	27	7	23	-

EX, emotional exhaustion; EF, professional efficacy.

The regression analysis showed that there was a statistically significant association between depression and treatment for a psychiatric illness in the last 3 months (*p* = 0.02). Participants with a current psychiatric illness were more likely to screen positive for depression (adjusted odds ratio [aOR] 4.06, confidence interval [CI] 1.28–12.91) than participants without a past psychiatric illness (Table 3).

**TABLE 3:** Regression model of depression scores against sociodemographic and clinical variables.

Variables	aOR	s.e.	95%	CI	*p*
Age	0.82	0.08	0.68	1.01	0.06
**Gender (Female)**					
Male	0.73	0.25	0.37	1.43	0.35
**Year of study (1)**					
2	2.83	1.94	0.74	10.85	0.13
3	3.60	2.68	0.84	15.51	0.09
4	1.94	1.53	0.41	9.11	0.40
5	3.63	2.86	0.77	17.01	0.10
6	2.16	1.98	0.36	12.96	0.40
**Income (<N$1000)**					
N$1001 – N$2500	1.08	0.73	0.29	4.09	0.91
N$2501 – N$5000	0.99	0.55	0.34	2.91	0.99
N$5000 – N$9999	1.20	0.69	0.39	3.69	0.75
>N$10 000	0.84	0.36	0.36	1.93	0.68
**Academic achievement: (>75 %)**					
<75%	1.12	0.56	0.42	2.98	0.81
**Has children: (No)**					
Yes	2.90	2.42	0.56	14.94	0.20
**Current psychiatric history: (No)**					
Yes	4.06	2.40	1.28	12.91	0.02
**Current medical illness: (No)**					
Yes	0.83	0.40	0.33	2.11	0.70
**History of COVID-19: (No)**					
Yes	1.36	0.47	0.70	2.67	0.37
**Current alcohol: (No)**					
Yes	1.40	0.44	0.76	2.59	0.29
**Current cannabis: (No)**					
Yes	2.66	1.72	0.75	9.41	0.13

aOR, adjusted odds ratio; s.e., standard error; CI, confidence interval; N$, Namibian dollars; COVID-19, coronavirus disease 2019.

Note: Content in brackets is the reference or base group.

Although bivariate analysis showed that there was an association between anxiety and gender (*p* = 0.02), the regression analysis showed that there was only a significant association between anxiety and treatment for a psychiatric illness in the last 3 months (aOR: 3.63, CI: 1.17–11.23; *p* = 0.003) (Table 4).

**TABLE 4:** Regression model of anxiety scores against sociodemographic and clinical variables.

Variable	aOR	s.e.	95%	CI	*p*
Age:	0.82	0.09	0.66	1.02	0.15
**Gender: (Female)**					
Male	0.71	0.25	0.35	1.43	0.19
**Year of study: (1)**					
2	3.06	2.17	0.77	12.24	0.10
3	3.95	3.06	0.87	18.01	0.04
4	1.77	1.47	0.34	9.06	0.42
5	3.19	2.65	0.63	16.25	0.24
6	2.00	1.96	0.29	13.70	0.39
**Income: (<N$1000)**					
N$1001 – N$2500	1.22	0.93	0.28	5.42	0.68
N$2501 – N$5000	2.25	1.31	0.72	7.05	0.13
N$5000 – N$9999	1.21	0.76	0.36	4.15	0.44
>N$10 000	2.06	0.96	0.83	5.15	0.08
**Academic achievement: (>75 %)**					
<75%	1.05	0.53	0.39	2.84	0.58
**Has children: (No)**					
Yes	1.07	0.94	0.19	5.94	0.55
**Current psychiatric history: (No)**					
Yes	3.63	2.09	1.17	11.23	0.003
**Current medical illness: (No)**					
Yes	0.93	0.44	0.36	2.36	0.80
**History of COVID-19: (No)**					
Yes	1.48	0.52	0.74	2.97	0.12
**Current alcohol: (No)**					
Yes	1.06	0.34	0.56	1.99	0.90
**Current cannabis: (No)**					
Yes	1.25	0.79	0.36	4.30	0.57

aOR, adjusted odds ratio; s.e., standard error; CI, confidence interval; N$, Namibian dollars; COVID-19, coronavirus disease 2019.

The regression analysis for burnout and the burnout domains is shown in Table 5. There was a statistically significant association between gender and EX (*p* = 0.01) and CY (*p* = 0.03). Older participants were less likely to experience high levels of EX (aOR: 0.75, CI: 0.61–0.93) and CY (aOR: 0.81, CI: 0.66–0.98) compared with younger participants. Male participants were less likely to experience EX (aOR: 0.40, CI: 0.20–0.79) and CY (aOR: 0.42, CI: 0.20–0.91) compared with female participants.

**TABLE 5:** Regression model of exhaustion, cynicism, and professional efficacy scores against sociodemographic and clinical variables.

Variable	Exhaustion					Cynicism					Professional efficacy				
	aOR	s.e.	95%	CI	*p*	aOR	s.e.	95%	CI	*p*	aOR	s.e.	95%	CI	*p*
Age	0.75	0.08	0.61	0.93	0.01	0.81	0.08	0.66	0.98	0.03	1.13	0.10	0.94	1.35	0.19
**Gender (Female)**															
Male	0.40	0.14	0.20	0.79	0.01	0.42	0.16	0.20	0.91	0.03	1.43	0.49	0.73	2.78	0.29
**Year of study (1)**															
2	1.02	0.74	0.25	4.24	0.98	1.81	1.50	0.36	9.17	0.47	0.70	0.47	0.19	2.62	0.60
3	1.40	1.11	0.29	6.67	0.68	0.99	0.83	0.19	5.15	0.99	0.35	0.26	0.08	1.48	0.15
4	1.13	0.93	0.22	5.71	0.88	0.88	0.76	0.16	4.81	0.88	0.83	0.64	0.18	3.72	0.81
5	1.50	1.24	0.30	7.53	0.62	1.97	1.70	0.36	10.73	0.43	0.46	0.35	0.10	2.03	0.31
6	3.37	3.39	0.47	24.27	0.23	3.23	3.40	0.41	25.42	0.27	0.15	0.14	0.03	0.90	0.04
**Income (N$0 – N$1000)**															
N$1001 – N$2500	0.94	0.65	0.24	3.64	0.93	1.02	0.84	0.20	5.12	0.98	0.48	0.32	0.13	1.79	0.28
N$2501 – N$5000	0.63	0.36	0.21	1.91	0.42	0.58	0.37	0.17	2.05	0.40	0.55	0.30	0.19	1.61	0.28
N$5000 – N$9999	1.67	1.05	0.49	5.73	0.41	1.20	0.86	0.29	4.91	0.80	1.51	0.91	0.46	4.93	0.50
>N$10 000	1.40	0.62	0.59	3.32	0.45	0.73	0.36	0.27	1.94	0.53	0.48	0.20	0.21	1.10	0.08
**Academic achievement (<75 %)**															
>75%	0.39	0.20	0.14	1.09	0.07	0.41	0.24	0.13	1.29	0.13	0.42	0.21	0.15	1.13	0.09
**Has children (No)**															
Yes	0.78	0.65	0.15	3.97	0.77	0.61	0.54	0.11	3.45	0.58	0.60	0.46	0.13	2.72	0.51
**Current psychiatric treatment (No)**															
Yes	2.94	2.07	0.74	11.67	0.12	1.35	0.95	0.34	5.36	0.67	1.58	0.81	0.58	4.34	0.37
**Past COVID-19 diagnosis (No)**															
Yes	1.20	0.45	0.58	2.50	0.63	0.85	0.34	0.39	1.85	0.68	1.42	0.49	0.72	2.79	0.31
**Current alcohol use (No)**															
Yes	1.53	0.52	0.78	2.97	0.22	1.54	0.57	0.75	3.19	0.24	1.05	0.33	0.57	1.93	0.88
**Current cannabis use (No)**															
Yes	2.30	1.81	0.49	10.79	0.29	6.34	7.42	0.64	62.97	0.12	0.72	0.45	0.21	2.46	0.60

aOR, adjusted odds ratio; s.e., standard error; CI, confidence interval; N$, Namibian dollars; COVID-19, coronavirus disease 2019.

Note: Content in brackets is the reference or base group.

## Discussion

This study, conducted shortly after the COVID-19 pandemic, showed that more than one-third of the medical students at UNAM screened positive for depression (43.6%) and burnout (36.2%). Just under one third screened positive for anxiety (30.6 %). The results of this study are of concern, because 77% of participants admitted to a cold negative attitude with a gradual detachment from their studies, while 68% of participants reported feeling extremely fatigued or emotionally exhausted. More than one in two students (58%) believed they were not up to the task.

In regression analysis, there was a significant association between depression (*p* = 0.02), anxiety (*p* = 0.003) and treatment for a current psychiatric illness. Furthermore, the final regression model also showed that there was a significant association between EX (*p* = 0.01), CY (*p* = 0.03) and female gender.

The prevalence of depression among medical students in this study (43.6%) is higher than that reported in previous studies from South Africa (36.4%),^33^ Uganda (21.5%)^34^ and Nigeria (15.1%).^35^ The prevalence is also higher than that reported in two international meta-analyses. Rottenstein et al. reported a prevalence of 27.2%^5^ while Puthran et al. a prevalence of 28%.^36^ However, the prevalence in this study is consistent with a regional meta-analysis that found that the prevalence of depression in medical students from Africa was higher (40.9%) than that in students from America, Europe, Asia, and the Western Pacific region.^6^ In keeping with the wide range (1.4% – 73.5%) in the prevalence of depression or depressive symptoms in medical students worldwide,^5^ the prevalence observed in this study was lower than that reported in Ethiopia (51.3%)^7^ and Egypt (65%).^37^

There is little information regarding the prevalence of depression in the general population of Namibia.^38^ Previous studies reported prevalence estimates ranging from 12.3%^39^ to 22.4%^40^ in selected population groups from Namibia. Taken together, this study confirms that the prevalence of depression in medical students from selected population is higher than in the corresponding general population.

A possible explanation for the high prevalence of depression among medical students in this study is that one third of the students in this study originated from families with a monthly income of less than 5000 Namibian dollars (N$) (equivalent to R5000.00 [South African Rands]). This is less than the average monthly household income of approximately N$6000.00.^41^ The average monthly wage in Namibia varies significantly depending on the sector and location. Monthly salaries range from N$4410.00 to N$77 900.00 with an average of N$17 400.00. Qualified medical practitioners are among the highest earners in the country with an average monthly wage of N$41 000.00.^42^ The fees at UNAM are approximately N$60000.00 per year and are therefore approximately 10 times higher than the average monthly income of some students’ families. It is therefore possible that pressure to secure funding for their studies, and high parental expectations contributed to the high prevalence of depression in this study. This is in keeping with the stressors identified in previous studies from Africa and other low- and middle-income countries (LMICs).^6,43^ It is also possible that student support services are less well developed compared with other medical schools, given that the UNAM medical school was only established 13 years ago.

The prevalence of anxiety in this study (30.2%) is in keeping with the prevalence among medical students from Africa (27.5%), the global prevalence (33.8%),^1^ and, is almost identical to that in a study from Ethiopia (30.1%).^7^ However, consistent with the wide range in the prevalence of anxiety internationally (6.8% – 88.8%),^1^ studies from other LMICs such as Brazil (37.2%),^44^ South Africa (45.9%)^33^ and Egypt (73.0%)^37^ reported a higher prevalence.

The prevalence of anxiety in this study was higher in pre-clinical students compared with clinical students and is in keeping with other studies from Ethiopia,^7^ Brazil^45^ and Pakistan.^46^ The prevalence of anxiety was lowest among students in their final year of study. Previous authors have suggested that this is likely to be because of the progressive adaptation to the environment and academic pressure as students advance through medical school.^45^

Although less well studied than depression,^15^ anxiety in medical students warrants further attention because it has been associated with impairment in physical^47^ and cognitive function.^48^ Anxious students were also reported to have poorer academic performance and less empathy for patients.^4^

There is significant overlap of symptoms between depression, anxiety, and burnout.^12,21^ The overall prevalence of burnout in this study (36.2%) is consistent with an international meta-analysis that estimated the global prevalence of burnout to be 37.2%.^49^

However, there is great variation in the prevalence of burnout from individual studies globally with estimates ranging from 7.0%^50^ to 95%.^51^ A meta-analysis reported that the prevalence of burnout in students from Oceania (55.9%) and the Middle East (53.7%) was higher than that in students from Asia (40.6%), Europe (27.5%) and South and Central America (26%).^2^ There is limited information from Africa, but studies from Africa confirm this wide variation in prevalence. Studies from Egypt (88%),^19^ and Uganda (54.5%)^8^ reported a prevalence that was more than five-fold higher than a study from Oman (7.0%).^50^

Therefore, a direct comparison between the prevalence noted in this study and the prevalence in other individual studies is not reliable because of several contextual factors. The overall prevalence of burnout is contingent on the criteria used to define burnout, the measurement instrument used, cut-off points and socio-cultural factors. For example, two studies that reported a prevalence higher than 90% used the Oldenburg Burnout Inventory (OLBI).^20,51^ The OLBI provides a score on the two sub domains only, without a formula for calculating the overall prevalence of burnout. In this study, the prevalence on the two sub domains of the MBI was almost double of that of the overall prevalence (77% and 68% vs. 36.2%).

It is also possible to use either a two- or three-dimensional model of the MBI to calculate the overall prevalence of burnout. In this study a three-dimensional model was used, and thus the overall prevalence of burnout was calculated utilising a combination of all three subscales of the MBI, that is, only students with a high score on the EX scale and a high score on the CY scale and a low score on the PE scale were included in the count for the overall calculation of the prevalence burnout. It is likely, therefore, that studies that used a two-dimensional model (usually high EX and CY) to calculate the prevalence would report a higher prevalence compared with this study.^52^

The final regression model also showed that there was a significant association between high scores on two of the subscales of the MBI and female gender. Although the regression model did not show that there was a statistically significant association between depression, anxiety, and gender, this study found a higher prevalence of depression and anxiety in females compared with males. Research has found that female students were more likely to score higher on neuroticism scales,^53^ and were more likely to suffer from psychological distress compared with their male counterparts.^4^ Previous studies confirmed that female students are at higher risk of burnout, because they are more negatively impacted by stressful life events,^37^ have other demands placed on them outside the university, and have less leisure time compared with male medical students.^54^ These findings suggest that specific interventions targeting female students may be needed at UNAM.

Unsurprisingly, there was a statistically significant association between students who screened positive for anxiety, depression and who were currently on treatment for a psychiatric illness. A previous study confirmed that students who were on treatment for depression or anxiety were at an increased risk of developing these disorders during their undergraduate studies.^13^

This study was conducted in May 2022, when all the students at the University of Namibia had returned to face-to-face teaching. However, COVID-19 restrictions were still in place in Namibia and the vaccination coverage in the country was only 23%.^55^ Previous studies have demonstrated that the COVID-19 pandemic led to poorer mental well-being in medical students.^11^ Continued concerns for their health, the risk of transmission from patient to student, and anxiety regarding the efficacy of online teaching during the pandemic may have contributed to the high prevalence of depression, anxiety and burnout in this study.

### Limitations

This study had several limitations. The cross-sectional study design means that no inferences can be drawn regarding causality. This study was conducted at a single medical school and the results are therefore not generalisable to other medical schools in Africa. The study did not collect data to quantify substance use. This information could have contributed to a better understanding of the association between substance use and depression, anxiety, and burnout in medical students. The study’s results may also be biased because students with depression or anxiety may not have participated in the study because of the fear of being stigmatised. Conversely, it is also possible that there was a selection bias, in that students who were depressed, anxious or burnt out were more likely to participate in the study, because they were more aware of their symptoms or because they were seeking help.

Nevertheless, this study has value because depression, anxiety, and burnout have significant adverse consequences during medical training and beyond qualification. Student psychological distress was found to impair students’ academic progress,^4^ professional development,^56^ and physical health.^23^ The cumulative effects of academic challenges and psychological distress were found to lead to higher rates of medical school dropout^22^ and even an increased risk of suicide.^3^ Furthermore, poor mental health among students while still at medical school has been shown to be a positive predictor of later distress in medical doctors after qualification.^57^ Substance use is a major public health issue worldwide, and studies have suggested that substance use in medical school may be the root of ongoing physician substance use.^58^

Besides these adverse outcomes for the individual student, physicians in Africa and other LMICs experience several other challenges as well. African public health systems are woefully underfunded, poorly resourced, and understaffed with a high disease burden. These factors were highlighted in a systematic review that confirmed a high prevalence of burnout among healthcare providers in sub-Saharan Africa.^24^ Depressed and burnt-out providers practising in a system with several organisational challenges will also be more likely to emigrate to developed economies, leaving behind a system that is not capable of providing the care people on the continent deserve.

It is thus important to determine the extent of the problem and to identify medical students who are at higher risk of these disorders. Although there is a wealth of literature on medical students’ well-being internationally,^1,2,4,6,36^ there is no information on the prevalence of depression, anxiety, or burnout among medical students in Namibia. In general, there is very limited mental health research from Namibia.^38,39,40^ A recent publication highlighted the lack of mental health research in Namibia, with calls for more collaborative research.^38^

This study, therefore, sought to understand the scope of the problem in the Namibia medical setting and fill a contextual gap in the literature.

## Conclusion

This study found a high prevalence of depression, anxiety, and burnout among medical students at UNAM. This warrants further interventions that prioritise the mental well-being of medical students. This study provides data to support the planning and implementation of student support services at UNAM. Given that the prevalence of depression and anxiety is higher in the first 2 years of medical school compared with the latter years, these programmes must be in place upon entry into medical school. Students need to be supported in an unfamiliar environment from day one of medical school.

The recommendations of the study are to identify students who are at risk of developing mental health problems early by raising awareness and educating faculty members about the symptoms of depression, anxiety, and burnout. To provide students with information on how and where to seek help. International efforts with structured wellness programmes have shown promising results.^59^ Thus, support services must be readily available and accessible. Furthermore, help seeking behaviour must be promoted at medical school level to prevent burnout in physicians. This study emphasises the need for longitudinal studies and more information regarding the mental health of medical students across Africa. Lastly, collaborative research must be initiated, and findings with other African researchers should be shared to protect a vulnerable group of individuals. It is hoped that the results of this study will be used to implement preventative and curative services that will improve the mental well-being of students at the university, and ultimately in the health workforce of the country.
